# The central role of national programme management for the achievement of malaria elimination: a cross case-study analysis of nine malaria programmes

**DOI:** 10.1186/s12936-016-1518-9

**Published:** 2016-09-22

**Authors:** Cara Smith Gueye, Gretchen Newby, Jim Tulloch, Laurence Slutsker, Marcel Tanner, Roland D. Gosling

**Affiliations:** 1 Malaria Elimination Initiative, Global Health Group, University of California, San Francisco, 550 16th Street, 3rd Floor, San Francisco, CA USA; 2 Independent Consultant, CRA 31A #9C-37, Champagnat, Cali, Colombia; 3 PATH, 2201 Westlake Avenue, Suite 200, Seattle, WA USA; 4 Swiss Tropical and Public Health Institute, Socinstrasse 57, 4051 Basel, Switzerland; 5 University of Basel, Basel, Switzerland

**Keywords:** Malaria elimination, Program management, Case-study

## Abstract

**Background:**

A malaria eradication goal has been proposed, at the same time as a new global strategy and implementation framework. Countries are considering the strategies and tools that will enable progress towards malaria goals. The eliminating malaria case-study series reports were reviewed to identify successful programme management components using a cross-case study analytic approach.

**Methods:**

Nine out of ten case-study reports were included in the analysis (Bhutan, Cape Verde, Malaysia, Mauritius, Namibia, Philippines, Sri Lanka, Turkey, Turkmenistan). A conceptual framework for malaria elimination programme management was developed and data were extracted and synthesized. Findings were reviewed at a consultative workshop, which led to a revision of the framework and further data extraction and synthesis. Success factors of implementation, programme choices and changes, and enabling factors were distilled.

**Results:**

Decentralized programmes enhanced engagement in malaria elimination by sub-national units and communities. Integration of the malaria programme into other health services was also common. Decentralization and integration were often challenging due to the skill and experience levels of newly tasked staff. Accountability for programme impact was not clarified for most programmes. Motivation of work force was a key factor in maintaining programme quality but there were few clear, detailed strategies provided. Different incentive schemes targeted various stakeholders. Training and supervision, although not well described, were prioritized by most programmes. Multi-sectoral collaboration helped some programmes share information, build strategies and interventions and achieve a higher quality of implementation. In most cases programme action was spurred by malaria outbreaks or a new elimination goal with strong leadership. Some programmes showed high capacity for flexibility through introduction of new strategies and tools. Several case-studies described methods for monitoring implementation quality and coverage; however analysis and feedback to those implementing malaria elimination in the periphery was not well described. Political commitment and sustained financing contributed to malaria programme success. Consistency of malaria programmes depends on political commitment, human and financial resources, and leadership. Operational capacity of the programme and the overall health system structure and strength are also important aspects.

**Conclusions:**

Malaria eradication will require adaptive, well-managed malaria programmes that are able to tailor implementation of evidence-based strategies, founded upon strong sub-national surveillance and response, with adequate funding and human resources.

**Electronic supplementary material:**

The online version of this article (doi:10.1186/s12936-016-1518-9) contains supplementary material, which is available to authorized users.

## Background

Global goals for malaria control, elimination and eventual eradication have evolved rapidly in the last year. A global goal of malaria eradication by 2040 was recently proposed [1], and at the same time, a new Global Technical Strategy for Malaria (GTS) was launched by WHO in 2015 and endorsed by all member states, providing the operational framework for achievement of elimination and stating an elimination goal of 35 countries by 2030 [2]. The overarching implementation and action framework, Action and Investment to Defeat Malaria (AIM) by Roll Back Malaria, was also launched in 2015 [3]. Many malaria programmes around the world are considering or committing to malaria elimination and are working to integrate the GTS and AIM principles into their national malaria programme strategy and framework. It is also likely that during this process, countries are considering the internal and external factors that may propel or impede progress towards elimination.

There are important challenges to address for both the long-term goal of global malaria eradication, as well as national elimination efforts. As highlighted in the GTS, countries must ensure political commitment and financing, and address major technical challenges, such as drug and insecticide resistance [2]. An overarching challenge at the national level is the inadequate performance of health systems. Deficiencies in health system structure may take the form of weak surveillance, inadequate tools for diagnosis and treatment, poor management of supply chains, an unregulated private health sector, weak monitoring and evaluation, and lack of adequate technical and human resource capacity. Ensuring that national malaria programmes have personnel with the appropriate level of programme management skills and tools to supervise and coordinate high quality implementation and evaluation is essential to achieving elimination, and, ultimately, eradication [2].

Today’s eradication goal is not the first effort to rid the world of malaria. The first attempt was made during the Global Malaria Eradication Programme (GMEP) (1955–1970). However, there are some major differences between that programme and present day efforts. The GMEP was based on vertical, time-limited interventions deployed through mainly centralized health systems, where authority was held mostly at the national level. In contrast, today’s health systems are mainly decentralized and malaria programmes are integrated into vector-borne disease control programmes [4]. While verticality brought some benefits, such as greater control and potential for motivation of staff, it also meant that activities were often not integrated with broader communicable disease activities, and lacked a clear, strategic component of surveillance with effective response packages, which created major challenges for achieving effectiveness and sustainability. Attrition of professional staff was increasingly a problem as GMEP progressed; the work became rote and routine and less about problem-solving. Without strategies in place to maintain motivation, trained staff left country programmes [4]. In some cases, national programmes following GMEP guidance did not adequately build up systems in country for capturing epidemiological data that could identify changing transmission patterns, or failed to evaluate the impact of interventions, leading to campaigns that became unable to reorient or adapt to changing contexts [4]. In addition, there was no agenda for research and development to accompany the GMEP. Therefore, as technical challenges such as drug and insecticide resistance arose, solutions were not forthcoming [5]. As financing for malaria eradication was withdrawn in the 1970s and 1980s, progress toward eradication stalled.

The lessons from the GMEP, as well as the framework of the GTS and the AIM and the new eradication goal, all speak to the importance of strong programme management as a central component for the success of countries aiming to achieve malaria elimination. The Eliminating Case-Study Series by the WHO Global Malaria Programme and UCSF Global Health Group was developed to detailed comparatively describe, analyse and discuss examples of national malaria programmes that are currently eliminating or have eliminated malaria. Thus, the case studies series offered an opportunity to review programme management strategies and contexts across countries to identify success factors along the road to elimination. In this paper, the authors report the findings of this cross case-study analysis, which is the first of its kind to examine countries in different socio-economic, political and ecological contexts. This analysis focuses on the way in which countries have implemented elimination programmes, have developed and adapted their malaria elimination strategies, and how they have operated within the context of different political, financial and human resources.

## Methods

This cross case-study review included nine of the 11 case-studies in the malaria elimination case-study series, produced through a collaboration between the WHO Global Malaria Programme and the Global Health Group, University of California San Francisco. Case-studies were included in the cross case analysis if they were in final English language draft at the time of analysis (November 2014). Case-studies included in this cross case analysis are Bhutan [6], Cape Verde [7], Malaysia [8], Mauritius [9], Namibia [10], Philippines [11], Sri Lanka [12], Turkey [13], and Turkmenistan [14]. Case studies from La Reunion and Tunisia were not included in this review because the report from La Reunion was not finalized nor translated into English at the time of analysis, and a draft of Tunisia was not yet available by the time the analysis was underway. Three of the nine case-studies represented countries in the prevention of reintroduction phase (Table 1), which have reached zero locally acquired cases and are actively preventing reintroduction of malaria [15].

**Table 1:** Case-study countries and elimination status

Country	BTN	CPV	MYS	MUS	NAM	PHL	LKA	TUR	TKM
Elimination status	Eliminating [6]	Eliminating [7]	Eliminating [8]	Prevention of reintroduction [9]	Eliminating [10]	Eliminating [11]	Eliminating [12]	Prevention of reintroduction [13]	Prevention of reintroduction [14]
Elimination history	Goal of zero transmission nationally by 2018; national malaria elimination certification by 2020	Achieved zero cases 1968–72 but epidemic occurred during 1977–79. Second elimination attempt 1983–85, however epidemic occurred during 1987–88. Goal of national elimination by 2020	Goal of national elimination by 2020: elimination in West Malaysia by 2015 and elimination in Sabah and Sarawak by 2020	First eliminated in 1969 and received WHO certification in 1973. Resurgence in 1975. Second elimination achieved by 1998	Goal of national elimination by 2020	Strategy of progressive sub-national elimination with national elimination (all provinces) by 2025 (recently updated to 2030)	Near elimination in 1963, then an epidemic from 1967–68. Zero local cases reported since November 2012; will seek WHO certification by end of 2015	Most of the country in consolidation phase in 1974, followed by epidemics in 1977 and 1993-1996. Last indigenous cases reported in 2012 during outbreak	First eliminated in 1961. In most recent attempt, the last indigenous case occurred in 2004. Received WHO certification in 2010

*BTN* Bhutan, *CPV* Cabo Verde, *MYS* Malaysia, *MUS* Mauritius, *NAM* Namibia, *PHL* Philippines, *LKA* Sri Lanka, *TUR* Turkey, *TKM* Turkmenistan

An initial conceptual framework for programme management in malaria elimination was developed to provide structure for the cross case analysis (Additional file 1). This framework was based on a document review of malaria elimination guidelines, reports, consultations and manuals to identify historical and current policy and research on management strategies, tools, and operational research. The document review took place in 2013 and 2014. The documents reviewed for the development of the initial conceptual framework were found using the following search terms: “program management,” “supervision,” “decentralization,” “vertical,” “integration,” “health systems,” “incentives,” “training,” “financing,” “costs,” “human resources” and “malaria,” “malaria control,” “malaria elimination” in Pubmed and Google Scholar (English only). A list of these documents can be found in Additional file 2. The framework was formatted in Excel as a matrix (Additional file 1). Using the framework components, two researchers (CSG, GN), reviewed each case study report for information (e.g., examples, synthesis or analysis) on program experience for each concept. If there were examples for a given concept, the experience was summarized in detail in the corresponding matrix cell. If there were no examples, the cell was left blank. After reviewing a given report across all concepts, a summary of the experience with a note as to how strong of an example it was (by subjective assessment) was written into the cell. After all of the reports were reviewed and cells filled in, main challenges and weaknesses of each programme experience were summarized by the researchers.

**Fig. 1:**
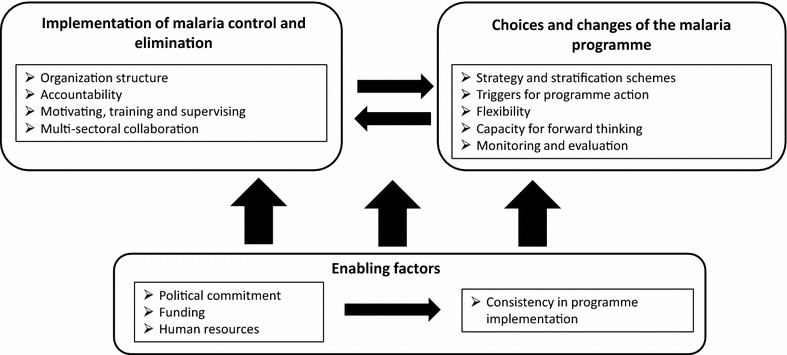
Final conceptual framework

A two-day workshop was held in 2014 to review the matrices on programme management and other themes. Malaria elimination researchers and experts conducted an in-depth review of case-study reports. Each reviewer read two reports and compared the information presented in the reports against the qualitative descriptions of experience, synthesis and analysis entered into the programme management matrix and summaries by CSG and GN to ensure that the data captured in the matrix were comprehensive, and to debate the lessons learned across the case-study experience. One of the results of the workshop was consensus that the framework needed revision to better capture the available data and draw firmer conclusions of major programme strengths and weaknesses. CSG combined the inputs from the workshop and additional documents collected (see Additional file 2) and re-reviewed to develop the new framework. The final conceptual framework for the analysis can be seen in Fig. 1. The framework was structured as follows: (1) Implementation-how malaria elimination is made to happen; (2) Malaria programme choices and changes; (3) Enabling factors, and how these factors affect the consistency of implementation. Using this new framework, CSG conducted a second round of in-depth review of the nine case-study reports, data extraction, summary and analysis. Ministry of Health, malaria programme personnel or other stakeholders were not interviewed for this analysis; however, data collection for the original case-study reports was based on extensive key informant interviews in addition to the quantitative data collection.

## Results

### Implementation

The ways in which malaria programmes were implemented were defined by several factors, including the level of decentralization and integration of the malaria programme, the health system in which the malaria programme operated its organizational structure and the accountability of the programme.

Decentralization is defined as the transfer of authority or dispersal of power and responsibility in public planning, management and decision making from the national level to subnational levels [16]. Most general public health programmes operated within a centralized structure until the early 1980s, when budget crises and recognition of inefficiencies led to widespread reforms [17].

Most of the malaria programmes in the case-studies operated within an integrated national health system. Integrated, or horizontal, programme service delivery is the delivery of services through the system of general health services [18, 19]. Vertical programmes, in contrast, are “directed, supervised and executed, either wholly or to a great extent by a specialized service using dedicated health workers”, an example of which is national smallpox eradication campaigns [18]. Most malaria programmes were integrated into curative health services provided by the national government, whereby malaria cases were diagnosed and treated in the national network of primary health care facilities. However the management and operations of the other malaria programme activities were often less clear. Some programme elements, such as surveillance and response approaches or prevention strategies through vector control, were conducted in a semi-vertical fashion by sub-national malaria-only units run by malaria regional officers or malaria technicians in basic health units. In other countries, integration with other vector-borne diseases translated to sub-national offices that coordinated vector control for all vector-borne diseases, using the same funding and personnel to conduct vector control for dengue, malaria and other diseases. Refer to Table 2 for a summary of the key learnings from implementation of malaria elimination programmes.

**Table 2:** Key learnings from implementation of malaria elimination programmes

• Most programmes operated in a decentralized health system, which in some cases led to greater engagement in malaria elimination by subnational health offices and communities • Most programmes were integrated, where malaria programme services were delivered through the system of general health services. Integration was overall a negative experience for most malaria programmes because staff were often given too many roles and responsibilities that were not clearly defined • During the early period of transition to decentralized and/or integrated programs, challenges were faced in maintaining quality and execution of interventions • Accountability for programme impact was not clear for most programmes • Motivation is important to maintain quality of interventions and different groups are and can be incentivized in different ways • Sustained capacity building and strong supervision are key to successful elimination • Working with other sectors to share information and develop and implement interventions has led to greater effectiveness in surveillance, prevention and targeting	

Table 3 shows the type of organization and level of integration of disease control within each country with estimated time frame of when these processes were underway. Table 3 also includes a measure of the clarity of accountability within each malaria programme, which was an assessment based on the information in the case-studies on the responsibility for progress or impact, decision-making, and funding flow structure of each malaria programme.

**Table 3:** Level of decentralization, integration and clarity of line of accountability affecting the national malaria programmes of the nine countries, with year of decentralization and integration if available

	Centralized vs decentralized health system	Vertical vs integrated malaria programme	Clarity of line of accountability as described in the case-study^a^ 0, +, ++
BTN	Decentralized since 1981 to district, further delegation from districts to subdistrict level beginning in 1990 and scaled up by 1996	Integrated with other vector-borne diseases since 2003	0
CPV	Decentralized to “health delegation” (local health authority) level	Integrated with other infectious diseases	0
MYS	Decentralized to the state level	Integrated malaria programme since 1981 (national) and 1986 (Sabah and Sarawak)^b^	+ Funding and decision making mainly originated from the central level, while the states were also held accountable for the impact on the ground
MUS	Decentralized	Semi-vertical malaria programme structure, malaria programme was integrated into the public health system in 1968	++ Semi-vertical malaria programme translated to most accountability resting with the malaria division of the Communicable Diseases Control Unit at the national level
NAM	Decentralized	Integrated malaria programme structure since inception in 1991^b^	++ National level appeared to be most accountable
PHL	Decentralized starting in 1958, implemented thoroughly in 1990s	Integrated malaria programme with health services since 1982, however some vertical elements (regional and sub-regional malaria specific positions) remain	++ Local level malaria programmes were relatively autonomous and accountable for the progress of malaria control, however there were nationally-funded personnel in each province to supervise and monitor activities but with no decision-making authority
LKA	Decentralized since 1989	Malaria is integrated with other vector borne diseases and with curative services through health system structure	++ National office appeared to be mainly accountable, however district malaria officers were responsible for malaria implementation and impact in their districts and reported to both the national programme and regional director of health services
TUR	Centralized system, Ministry of Health responsible for health care and social welfare activities, supervises all medical and health care personnel in the public sector, Education and health services are provided by the central government	A vertical malaria network was developed since 1920s with three levels: 1. National Malaria Commission (national level); 2. Province/district level with laboratory and headed by a physician with staffing of other malaria control personnel; 3. Peripheral level (subsections or “circles” of 10–15 villages), with personnel for vector control	++ The Directorate of Malaria Control was accountable for malaria strategy and achievements
TKM	Not clarified in case study, but assumed to be centralized	Most likely semi-vertical, The Sanitary Epidemiological Service (SES) responsible for communicable disease control including anti-malarial interventions, national, provincial and district level SES offices, SES considered specialized in malaria control, and works with the general primary health care services for malaria interventions	++ National-level SES appeared to be accountable for the impact of the malaria programme

*BTN* Bhutan, *CPV* Cabo Verde, *MYS* Malaysia, *MUS* Mauritius, *NAM* Namibia, *PHL* Philippines, *LKA* Sri Lanka, *TUR* Turkey, *TKM* Turkmenistan

0 not clear, + moderately clear, ++ clear

^a^The measure of the clarity of accountability within each malaria programme was an assessment based on the information in the case-studies on the responsibility for progress or impact, decision making, and funding of each malaria programme

^b^No further information provided in case-study report as to the type of integration of the malaria programme

In addition to the role of integration, decentralization and accountability, implementation was also impacted by the motivation package for malaria workers, structure of incentives, training programmes, and supervisory structure offered by malaria control programmes. The role of intersectoral collaboration and its impact on malaria elimination was further explored.

#### Impact of decentralization on malaria programmes

Overall there were numerous examples of the decentralization process creating challenges for the implementation and quality of malaria control, with possible contribution to increases in incidence during the transition period when decentralization was first implemented. However, once in place, and roles and responsibilities were clearly assigned, in most cases decentralization increased local level capacity of the malaria programme as well as community access to health services. Across the case-studies, vertical programmes operating in a centralized health system (such as Turkey and Turkmenistan) appeared to have the clearest lines of accountability and assignment of roles and responsibilities, as there was one level responsible for both elimination strategy development and outcome measurement. However, in more complicated settings, some case-studies clarified well which office or person was ultimately accountable for programme operations and impact (Table 3). In Malaysia, decentralization was seen as benefiting elimination. While the malaria programme at the national level developed policy, provided technical expertise, and controlled the finances, each state and district had a vector-borne disease programme office which managed, coordinated and implemented malaria activities. The programme engaged state offices in the elimination planning by holding elimination workshops. Additionally, the malaria programme believed that the development of sub-sector offices, or malaria offices in remote localities in Sabah State had a substantial impact on reducing malaria morbidity by facilitating integration of the malaria programme into communities. Through engagement with the local level, there was a greater level of accountability sub-nationally for implementation of the malaria programme and its impact on the ground. However, funding and decision-making appeared to mainly rest with the national level.

In contrast, decentralization in Cape Verde appeared to impede malaria programme progress, mainly because capacity was low at the health delegation (local health authority) level and there were few resources or capacity for the central malaria programme to supervise or implement activities in the peripheral areas. Accountability for the quality and impact of programme activities was not clear.

Decentralization led to both positive and negative outcomes for the malaria programmes. In the Philippines, the process of decentralization led to a seemingly chaotic malaria programme environment in the 1980s and 1990s. At certain points, provincial and municipal level authorities were not able to lead malaria control efforts because of insufficient training and resources. There was a high degree of variation across provinces in quality and extent of implementation, NGO involvement, and external funding. In Laguna Province, for example, devolution in malaria free areas disrupted progress as malaria personnel were reassigned to other activities. This led to a disruption of the surveillance-response system and created a vacuum of experience when an outbreak occurred, which then threatened to spread into other receptive, malaria-free provinces where capacity was also inadequate. However, the positive impacts of decentralization included an increase in provincial, municipal and community ownership of malaria, eventual growth in staff and skills based in the field and a tailored approach to malaria control and elimination. Department of Health (national level) staff were positioned in each province to supervise and monitor activities, but they were not given decision-making authority. Local, sub-national staff appreciated this autonomy and turned to higher levels only when in need of technical guidance.

Bhutan’s decentralization process was also relatively disorganized in the early years, when districts began managing the delivery of basic services. Malaria incidence rose from 5213 cases in 1983 to 18,368 in 1984. The increase in cases was believed to be the result of a decline in indoor residual spraying (IRS) coverage and quality. However, once the process was more established, decentralization may have contributed to longer term reductions in malaria cases through an expansion of facilities and deployment of malaria workers in health centers in endemic areas, boosting surveillance activities.

In Sri Lanka, decentralization may have also contributed to an initial rise in malaria-related incidence and deaths. From 1990–99, when the system was undergoing the transformation, confirmed infections rose from 142,294 (1995) to 264,549 (1999). Then, from 2000 onward, incidence declined. The national programme formulated malaria control policy, monitored national malaria trends, provided technical guidance, and undertook entomological and parasitological surveillance. District-level offices coordinated parasitological and entomological surveillance, vector control, and conducted supervision and M&E activities. Even with the initial challenges, decentralization may have contributed to stronger leadership at the district level and local adaption of the programme. Accountability mainly rested with the national office; however, district malaria officers were also held accountable to the national programme.

Turkey’s centralized health system may have contributed to the consistent approach of the public health services, and the maintenance of skills, capacity and malaria control activities over time. Challenges included staff shortages for active case detection in certain parts of the country. Staff were often transferred from areas where transmission had been interrupted to areas with current transmission, leaving malaria-free areas vulnerable in the event of resurgence.

Turkmenistan’s centralized health system may have also led to a greater degree of consistency in the programme, accountability at the national level, and political support. However there were challenges in the programme, including delays in diagnosis, treatment and reporting in rural areas.

In Namibia, the national malaria programme provided funding, trainings and commodities to the regional level, which coordinated district activities. Due to the fact that donor funding for malaria almost exclusively moved through the national programme, accountability rested mainly at the national level.

The Mauritius case-study described its malaria programme as semi-vertical and the bulk of malaria personnel sat in the national office. Accountability for programme implementation and impact rested mainly at the national level.

#### Impact of integration on elimination progress

The impact of integration on malaria control efforts was overall considered negative, mainly due to overburdening staff with often-undefined roles and responsibilities. There was also a risk that the integrated programme would dedicate fewer resources to malaria when cases were reduced. The potential positive outcome of increased programme efficiencies and cost savings through integration (e.g., with all vector-borne diseases) was not documented in the case-studies.

Bhutan, Sri Lanka and Cape Verde provided clear examples of malaria programme integration with the transition from single-disease to multi-purpose technicians. In Bhutan and Sri Lanka the impact of integration was just occurring at the time of data collection and its impact was not described. Before integration, malaria technicians in Bhutan posted in hospitals and basic health units were responsible for all malaria diagnosis, treatment, and parasitological and entomological surveillance and vector control. Sub-national Health Delegates in Cape Verde became overloaded as they were responsible for clinical and administrative duties in addition to supervising the team of health technicians responsible for coordinating interventions and surveillance.

In Mauritius, the immediate integration of malaria control into the general public health services once the last indigenous case was detected in 1968 was likely a contributing factor in the resurgence of cases in the 1970s. Medical officers were newly responsible for malaria in their districts. These rapid transitions contributed to poorer quality passive case detection, lack of participation by health workers in malaria screening programmes, and financial constraints after integration.

In Namibia, integration similarly presented logistical and bureaucratic hurdles: one example was that drivers were not available for malaria related activities because they were busy with other Ministry-related tasks.

Malaysia’s integration of the malaria programme occurred after 1981, when elimination was declared infeasible. The malaria programme was integrated with other vector borne diseases at the central and state-based offices. Since dengue was the most common notifiable disease in 2010, it is likely that integration diverted resources away from malaria programme activities.

Turkey provided a contrasting example of a vertical malaria programme. It appeared that the programme’s verticality led to a greater level of consistency in programme activity and resources, and translated to clear accountability at the national level for malaria strategy and achievements. However, during the epidemics recorded in the 1970s and 1990s, a contributing factor was waning interest in malaria control as the caseload became very low. It is theoretically possible that an integrated programme would been more flexible and cost-effective, able to shift staff or resources as needed across programmes.

The Philippines and Turkmenistan reports were not specific about the level of integration of the malaria programme.

#### Motivation and incentives

Maintaining a high level of motivation in implementers is important to sustain consistency and quality of interventions. Staff motivation is an important aspect of human resource capacity, and depends on a number of factors: working conditions, financial incentives, correct and prompt compensation, management of staff and possibilities for professional advancement [20]. This basic motivation package is often the key to a successful malaria programme. As malaria cases decline, different strategies must be employed to keep staff committed, and prevent turnover and loss of institutional knowledge. Incentives may be used if specific and predetermined milestones are achieved. Incentives are the “rewards and punishments that [service] providers face as a consequence of the organizations in which they work, the institution under which they operate and the specific interventions they provide” [20]. Community level and political motivation is also a key predictor of malaria elimination success, and can be significantly enhanced by including elimination targets and legislation. The case-studies described structures and mandates that motivated and incentivized communities or other sectors to engage in malaria elimination. Professional incentives were in place for volunteer health workers, but not described for malaria programme workers.

In Sabah State of Malaysia, Primary Health Care Volunteers (PHCV) were motivated by the prospect of professional work. PHCVs played an important role in diagnosis, treatment and prevention. IRS spraymen were often recruited from the PHCV pool, offering the possibility of a future paid position. Similar to the Sabah programme, the Philippines used incentives for the work of the paid Barangay (i.e., village) Health Workers. In addition to earning a small wage and an important role in the community, their microscopy skills and responsibilities were highly regarded among the primarily uneducated women who participated in the programme, who otherwise had limited employment opportunities.

The structure of external grant funding in the Philippines inadvertently led to a shift in non-financial as well as financial motivation in municipal staff. When the Global Fund grant support began in Apayao Province, the grant was structured such that municipality offices were given responsibility for planning and management, which translated to an increased level of motivation of municipal staff to take initiative and ownership of malaria control, with the positive affect of an increase in level of confidence for managing the malaria programme activities.

External grant funding increased the level of motivation to conduct comprehensive field work in Sri Lanka, because that funding covered travel costs and per diems for intensive entomological surveillance and supervision. However, delays in payment for the overtime or traveling claims counteracted the potential beneficial impact of these incentives.

The Sabah State malaria programme’s collaboration with private sector plantations (e.g., palm oil plantations) showed the motivating factors for plantations to get involved in malaria control. Plantation owners provided malaria diagnosis and treatment and some also provided vector control (either directly or through support to the district malaria programme office) because they believed the benefits of the collaboration to be an increase in plantation worker productivity and reduction in worker health care costs, an increase in their profile as a good place to work by providing access to health care and prevention on site, and abiding by expectations in Malaysia for corporate social responsibility and adherence to the labour laws.

Mauritius programme staff considered their public health laws enforcing environmental management and access to homes to conduct vector control to be motivating factors for community acceptance and participation in the malaria programme’s vector control activities.

Certification schemes kept motivation at higher levels in Turkmenistan and the Philippines. Turkmenistan created a certification for laboratory quality, for which every laboratory participated in a scoring system for diagnosis, and labs exceeding an 80 % score received a 1-year certification. One example of an overarching elimination-friendly structure is the Philippines subnational elimination certification process, where provinces were reviewed and validated as “malaria free.” This structure was thought to have motivated provincial staff and community participation in malaria control.

Bhutan and Cape Verde did not report on incentives. Namibia’s programme found difficulty in recruiting support to and engagement of communities because their health volunteers were not motivated through financial or professional incentives.

#### Training

Training is an important component of a malaria elimination and POR programme. While there was very limited information in the case-studies on the coverage and consistency of trainings, several country experiences showed the risk of weakening the malaria programme when training programmes became infrequent or inadequate. Conversely, programme activities were strengthened when training was increased with the injection of external funding or if an outbreak occurred. The content and format of trainings were not well described and appeared to be weakly structured in the case-studies, and there was very little information on surveillance and response training. Most programmes did not describe on-the-job training formats, so it is assumed that they were large, seminar-style trainings.

There were several countries with inadequate training programmes and resultant operational challenges. Because training in Namibia was insufficient, quality of diagnosis and treatment services, record keeping, coordination, time management and communication were suboptimal. These problems were exacerbated by poor job descriptions. In the Philippines, lack of training and follow up when a new drug policy was rolled out meant that some staff were unaware of the new policy and how to implement it. Inadequate surveillance training in Turkmenistan weakened the programme in the 1990s and contributed to the outbreak of 1998–99. The problem was exacerbated by understaffing, in that many Russian specialists left the country after independence in 1991. Post outbreak, the programme increased capacity with the development of a continuous education programme (2004–10), reaching more than 1400 personnel. In 2010, 11–20 % of the government malaria budget was spent on staff training.

Global Fund grants provided funds to increase the number of trainings in Bhutan, Philippines and Sri Lanka. Sri Lanka held regular trainings to maintain engagement in elimination. Philippines’ trainings led to improvements in planning and delivery of interventions.

Bhutan, Cape Verde, Malaysia, Sri Lanka and Turkey prioritized microscopy training to ensure that diagnosis skills were maintained as malaria cases declined.

#### Supervisory structure

Supervision of malaria control activities is conducted to ensure the quality of interventions and to increase or maintain a high level of motivation in the workforce [21]. Effective supervision should include regular visits to the periphery and activities to check information and supplies, problem-solving with the employee, and a feedback mechanism to encourage improvement [21]. In the case-study reports, supervision of intervention quality, timing, coverage and measurement of impact were not described. Resource constraints in some programmes limited staff time and transportation to carry out supervision of field activities.

In some case-studies the planned (though not necessarily executed) supervision structure was reported. In Bhutan, the malaria technicians supervised village health workers and health centers. The Sanitary Epidemiological Service (SES) of Turkmenistan, which was overseen by the Deputy Minister of Health, monitored coverage and performance of all interventions. In both Bhutan and Turkmenistan, the quality assurance process and supervision of microscopy were highlighted as key activities.

In the Philippines, Department of Health representatives at the provincial level were reluctant to supervise as the local staff worked autonomously. Thus, provincial authorities viewed health workers and local staff as partners and supervision in the traditional sense did not occur.

The Namibia and Sri Lanka programmes provided timeframes for supervision. In Namibia, national staff planned to conduct annual regional supervisory visits, regional staff conducted quarterly district visits, and district staff visited clinics on a monthly basis. Sri Lanka’s Regional Malaria Officers aimed to visit all district entomological surveillance, IRS programmes, and up to two active case detection activities each month.

In Sabah State of Malaysia, state and district level entomologists supervised insecticide treated net (ITN) and IRS programmes conducted by district and sub-district offices. The national level laboratory supervised all microscopists and provided retraining for those who committed frequent mistakes. District level malaria officers supervised health volunteers.

In Mauritius, IRS activities by public health staff were “strictly” supervised by the team. IRS was conducted for 2 years within 500 m of a case’s residence.

In some countries, resources were not sufficient to maintain adequate supervision. Lack of transportation and limited staff in Sri Lanka cut down the amount of field supervision in recent years. There was little funding or time available for Cape Verde’s 17 Health Delegates to supervise the network of primary health facilities and health technician teams. In Namibia, a supervisory structure existed but was not followed due to time constraints and unreliable transportation. In Sri Lanka and Namibia, the injection of Global Fund grant funding increased the resources for supervision.

#### Multisectoral collaboration

The case-studies provided examples of multisectoral collaboration, where malaria programmes worked or planned to collaborate either with other Ministries in the government, such as the Ministry of Labour or Foreign Affairs, or other, non-health sectors including private sector health facilities or extractive industries. This type of approach is different from programme integration with national curative or preventive services, but can work well within that structure. Resurgences in several countries may have been prevented by identifying risks, sharing information and collaborating on malaria education and control with other ministries and the private sector. Contracting out services to private entities, which was done by some countries, may be cost-effective and increase quality and coverage. There were no experiences documented of programmes working with small enterprises or local chambers of commerce.

The Bhutan malaria programme reported a need to involve other Ministries because of economic development activities and related migrant labour entering the country. Cape Verde developed collaborations with the Ministry of Agriculture and Environment, to improve agricultural practices and the management of rural water sources.

Some programmes outsourced malaria control implementation to NGOs. Namibia and Angola launched the Trans-Kunene Malaria Initiative (TKMI) in 2009 to monitor cross-border importation and coordinate communication, activities and policies. An NGO provided the funding and support for long-lasting insecticide-treated net (LLIN) distribution and monitoring along the border area. An NGO in the Philippines became the Principal Recipient of the Global Fund grant in funded provinces. In Sri Lanka, LLIN distribution in the conflict-affected areas of the country relied upon local NGOs, as they had community based ties to the region and were able to operate in areas that presented major challenges for the malaria programme.

Several case-studies presented examples of collaborations with private sector plantations or construction companies. During its first elimination campaign, Mauritius collaborated with private sugar companies to educate employees about malaria prevention and to create private clinics for screening and case reporting. More recently, the Sabah State malaria programme in Malaysia developed informal partnerships with private sector plantations. Plantation owners contributed resources by building subsector offices or clinics for malaria programme workers and contracting out or conducting IRS for plantation structures. Leaders of Apayao Province in the Philippines worked with local mine and mill operators and dam construction companies to identify projects that would increase the flow of migrant labour in high malaria risk areas and to develop joint screening and reporting systems. In Turkey, the malaria programme monitored irrigation systems.

Turkmenistan had a similar challenge, where there was an increase in vulnerability to malaria transmission due to military training activities and migrant worker mobility, both of which contributed to the outbreaks of malaria in 1998–99 and 2002–2003. Vulnerability is the proximity to malarious areas or resulting from the frequent influx of infected individuals or groups or infective anophelines [15]. In response, in 2005, the Chairman of the Cabinet of Ministers of Turkmenistan approved several documents calling for intersectoral collaboration and implemented plans including collaborating with the Ministry of Defense to share information, immigration services, and construction and oil companies that import labor to provide health information, free access to diagnosis and treatment.

### Malaria programme choices and changes

Most case-studies documented the development of strategic plans and strategies for targeting interventions. Some programmes showed a high degree of flexibility and forward thinking. Surges in action by programmes were often reactionary and not a result of robust planning, and there were gaps in many programmes in monitoring and evaluation, particularly in the provision of feedback to the lower levels of the health system. Table 4 provides key learnings for programme choices and changes.

**Table 4:** Key learnings from malaria elimination choices and changes

• Strategic plans and stratification strategies are an important part of programme planning • In most cases, programme action occurred as a result of an increase in malaria cases or deaths. In a few cases, a new elimination goal or leadership drove action • Some programmes showed a high degree of flexibility and adoption of new strategies and tools • Programmes likely avoided outbreaks by working with other sectors to identify and respond to threats of importation or increased incidence • Analysis and feedback of programme performance to the periphery is important but not well-described in the case-studies	

#### Strategic plans

The case-study reports had limited information on programme strategies and activities, and how they were designed. Five case-studies reported on the national strategic plan objectives and planned interventions (Bhutan, Cape Verde, Philippines, Turkey and Turkmenistan). It is assumed that all countries had strategic plans for control and elimination, but these plans were not described in the case-study. Most reports did not indicate whether the specific strategies were followed, or whether there was 100 % compliance in coverage and timing. This is discussed in the M&E section.

#### Stratification

Stratification plans assist malaria programmes in targeting interventions and managing threats of increased receptivity or vulnerability. Receptivity means there is presence of anopheline vectors and existence of other factors favoring malaria transmission [15], while vulnerability describes the risk of malaria importation. Stratification plans may be based on disease epidemiology, entomological data, and socio-economic and development factors, such as development projects or population movement. Five case-studies included a recent stratification system (Table 5). Although the spatial scale and categories used in the stratification systems varied greatly, these systems identified priority areas for programmes to dedicate resources and response activities. Overall, a major gap in stratification plans across the case-studies was the surveillance and response strategies developed to mitigate the threat of vulnerability.

**Table 5:** Stratification systems, last year of update, and spatial scale

	BTN	CPV	MYS	MUS	NAM	PHL	LKA	TUR	TKM
Listed stratification system in case study	Yes (classification + activities)	No	Yes (classification only)	Yes for period 1979–1982	No	Yes	No	Yes	No, but information on type of foci classification and response detailed
Last year of updated stratification system	2012	No info	2008, was planned for 2013	No info	No info	1996, 2010, 2013	As of 2010, stratified case based interventions to be designed	In place from 1977 onwards (through to publishing of case study)	Focus register established and updated (2004–2010)
Spatial scale	District	Foci have not been “properly explored, delimited and classified”	“Locality” (e.g., village, plantation section, or housing area)	“Locality” in which at least one local case had been detected (1975–1981)	Region	Provinces, based on indicators at barangay (or village) level (e.g., number of barangays with cases)	Presumably by district	Strata are a collection of provinces. Foci also used, and defined as minimum unit of anti-malarial activities. Focus registered maintained at province level	Focus, Minimum unit of malaria control action (e.g., one settlement)

*BTN* Bhutan, *CPV* Cabo Verde, *MYS* Malaysia, *MUS* Mauritius, Namibia, *PHL* Philippines, *LKA* Sri Lanka, *TUR* Turkey, *TKM* *NAM* Turkmenistan

The spatial scale used in the stratification systems varied across the countries. Some countries used the WHO foci classification system, which is currently comprised of seven categories of transmission foci (new potential, new active, endemic, residual active, residual non-active, cleared up, pseudo focus) [15]. The POR countries of Mauritius, Turkey, and Turkmenistan reported using the foci system. Bhutan used a district-level stratification system, but in 2012 adapted the foci-based system using six categories (endemic, residual active, residual non-active, new active, new potential, cleared-up). Mauritius used three categories (active residual foci; active new foci with more than three cases; active new foci with less than three cases). Other countries employed different spatial or descriptive scales. “Localities” in Malaysia were described as a village, section of plantation or housing area. Other countries used district or regional administrative units. In the Philippines, the unit of administration was the province, but the category of risk was defined at the barangay (village) level and aggregated up to the province level.

Only the Malaysia and Turkmenistan reports described how the programmes developed their stratification system. Malaysia reviewed incidence over 3 years, vector receptivity, and access to the health system. Turkmenistan reviewed case investigation and surveillance data combined with the malaria foci record “passports”, which included mapping. Programmes updated their stratification systems from every 3 years up to every 30 years or more.

The stratification strategies in Mauritius, Philippines and Turkey outlined response activities to be applied in each strata. The malaria programmes with clear stratification criteria, indicators and vector control targets appeared to benefit from these systems. Stratification was viewed as extremely helpful by the Philippines programme and in Turkey was believed to have facilitated appropriate decision making for the application of vector control. Namibia’s stratification system allowed for targeting of resources to priority areas; however the risk of importation and onward transmission in some areas was likely underrepresented. In Sri Lanka, epidemic forecasting based on entomological surveillance identified areas for mobile surveillance clinics and focal IRS.

#### Programme action

Some malaria programmes were spurred to action in response to increasing transmission, an outbreak or an increase in mortality. In most cases, these events were caused by waning surveillance or control, precipitated by a weakening of the surveillance and response system, which subsequently required a ramp up of resources and programme intervention. In other cases, surges in action were the result of leadership at the national or subnational level.

In several countries, a gradual decline in programme efforts led to outbreaks, which in turn encouraged programmatic action. Turkey did not plan for potentially increasing transmission when major irrigation projects were underway and increases in migration were expected. These factors contributed to an outbreak in 1977. In response, the programme was given a larger allocation by the government, surveillance agents and microscopists were hired, and laboratories received more funding. Turkmenistan saw an increasing level of vulnerability to malaria importation along the border with Afghanistan, and this risk was compounded by delays in diagnosis, treatment and reporting of malaria cases in some rural areas. These factors led to an outbreak in 1998–99, the response for which was an immediate increase in consumables for diagnosis and chloroquine and primaquine for radical treatment and chemoprophylaxis. A reduction in transmission followed. In Cape Verde, multiple outbreaks have occurred after IRS was reduced or halted; each outbreak triggered a surge in IRS coverage. The efforts, though, were not maintained as evidenced by the locally acquired cases recorded nearly every year. In 2006, an increase in mortality to eight deaths led to an investigation by the Cape Verde Ministry of Health and development of a new national goal of malaria elimination by 2020. In Malaysia, outbreaks in plantation areas of Sabah State stimulated several inter-sectoral private–public partnerships with plantations which included surveillance and vector control activities.

In several countries, endorsement of an elimination goal by government leaders led to programme action. When malaria elimination was adopted in the 1940s and re-adopted after its resurgence in 1975, the Government of Mauritius organized a military-like offensive. Philippines provided a similar example, but in a highly decentralized context, where in certain provinces motivated leaders augmented malaria screening and other control efforts by increasing staff and funding for the activities. In Namibia, action was spurred by the leadership of the former Minister of Health.

In some countries, programme activities continued consistently, without such surges in action. After experiencing resurgence in the 1970s, Sri Lanka maintained entomological and parasitological surveillance activities, which informed programme activities and response strategies. Bhutan also had relatively consistent programme implementation.

#### Flexibility of malaria programme strategies and approaches to problem-solving

Several countries showed a high level of flexibility by introducing new or adapting strategies, from insecticide rotation to lessen the risk of insecticide resistance, to an increase in parasitological screening in development areas to curtail the risk of transmission, to collaborations with the private sector. The case-studies did not have detailed information on the process undertaken to evaluate or adapt new strategies.

Sri Lanka, Bhutan and Malaysia showed evidence of flexible programming. Sri Lanka introduced a series of programme changes in the 1990s, including the introduction of targeted IRS to replace “blanket”, or universal, spraying, partly in response to new WHO recommendations [22]. IRS insecticide rotation was introduced in 1998, for which different types of insecticides were used in bordering districts with rotation of insecticides across districts over time in order to lessen the risk of resistance. Farmer Field Schools were developed in the late 2000s, building awareness about the connection between insecticide use for agriculture and for disease management. Then, between 2000 and 2011, primaquine for radical cure of *Plasmodium falciparum* infections and RDTs for mobile malaria clinics were rolled out. In 2009, as the country progressed toward elimination, a series of changes to its parasitological surveillance programme were introduced. Bhutan similarly instituted a series of surveillance and response activities, including parasitological surveillance, mapping and response measures starting in 2013.

The malaria programme of Sabah State of Malaysia also showed flexibility. In the mid-1990s, Sabah State responded to the need at the community level for better access to diagnosis and treatment by recruiting and training primary health care volunteers. In 1995, volunteers collected 14 % of the blood slides taken in the state.

Adoption of an elimination goal spurred the design and scale up of surveillance activities and integrated vector management (IVM) in Turkey, to be used in active foci and in emergency situations. The Ministry of Health supported these new strategies through decrees, regulations and guidelines.

Turkmenistan showed adaptive capacity in its response to two outbreaks that occurred post-elimination. A suite of epidemiological, treatment and entomological surveillance interventions and policies were rapidly executed in response to the 1998–1999 outbreak, along with monitoring and supervision by the national programme. However another outbreak occurred within 3 years (2002–2003), meaning that these measures were likely not sustained. The outbreak of 2002–2003 led to a similar scale up of interventions and surveillance, and also led to sweeping changes to strengthen the whole malaria control system starting in 2004.

The programme in Mauritius appeared to stay consistent over time. During the period of resurgence, the programme showed flexibility in that it was able to mobilize a large number of staff. The Cape Verde and Namibia case-studies did not contain specific examples of flexibility in the malaria programme.

#### Programme capacity for forward thinking

Malaria programmes in the case-study series did not show a high level of capacity for anticipation of threats to elimination. There are many examples of major development projections that combined a potential for increased receptivity and vulnerability. In general programmes either did not identify these challenges in advance therefore did not have adequate or timely responses to these threats, or they were unable to secure funding to mitigate the impact. In either case, there appeared to be a lack of coordination with other sectors. In the few examples where programmes did work with other sectors, it appeared to help avoid outbreaks or epidemics.

Several malaria programmes did not anticipate the increased risk of malaria transmission posed by irrigation and reservoir projects. In Sri Lanka, dam construction and forest clearing for rice cultivation in the malaria-endemic eastern part of the country likely increased receptivity and led to the epidemic of 1987. There was no documented action by the malaria programme, nor was there evidence in the case-study that resources were increased, to offset these risks, and the epidemic grew to 687,599 cases by 1987. Reservoir construction in the 1980s and 1990s in Turkmenistan also led to increases in receptivity as the filtration ponds increased anopheline breeding habitat. At the same time (1980s), the dissolution of the Union of Soviet Socialist Republics (USSR) increased population movement into the country from Afghanistan, where malaria endemicity was higher. These developments contributed to the outbreaks in the 1990s.

The epidemic of 1977 in Turkey, which took place only 3 years after the country reported its last indigenous malaria case, was caused in part by an extensive irrigation project which increased breeding habitat and the level of internal migration from higher transmission areas in the eastern part of the country. However, the programme did not increase its surveillance efforts and by 1977 there were 115,512 recorded *Plasmodium vivax* cases. A second epidemic occurred in 1991, even though the irrigation canals were covered to prevent mosquito breeding. The programme did not adequately respond to large-scale internal migration from remote, endemic rural areas into the irrigation project zone, nor account for the flow of migrants from neighbouring countries, many of which were not politically stable and some of which were experiencing *P. vivax* epidemics. There were an inadequate number of malaria personnel in the affected areas and it is unclear whether additional resources were made available to the programme to stymie the risks. Subsequently, there was an increase from less than 15,000 cases in 1991 to more than 84,000 by 1994.

When an influx of construction workers entered Mauritius after a major typhoon, a resurgence of malaria occurred from zero local (since 1968) to 41 cases in 1975. Based on this experience, Mauritius anticipated future risk of imported malaria by implementing an extensive border screening programme, including follow up visits and screening for arriving at-risk groups and detected positive cases. Similar to Mauritius, an increase in intra-national and international movement in Cape Verde occurred as a result of improvements in ports and airports. The programme did not anticipate the impact of these projects. Bhutan’s malaria programme was rapidly moving toward elimination and was keenly aware of the high receptivity and vulnerability along the southern border with India. In addition, there were an estimated 35,000 documented workers in the country, the majority of which were employed in large-scale development projects in the interior and southern districts. Proactive case detection started in these development zones where migrants live and work. The programme also piloted Community Action Groups in the southern, receptive districts that have larger migrant flows from India. These groups enlisted community support in prevention and control of malaria.

The number of migrant workers originating from high transmission neighbouring countries propelled Malaysia’s Sabah State to begin collaborations with private sector plantations in the early 2000s to implement and surveillance of malaria to anticipate and avoid outbreaks.

The Philippines planned for malaria elimination by developing a certification process for subnational elimination in 2011, believed to be necessary because of the geography of the country, which is composed of many islands with different malaria potential.

The Namibia case-study did not provide specific examples of anticipation of threats.

#### Monitoring and evaluation

Monitoring and evaluation (M&E) includes monitoring programme outputs, such as whether intervention coverage and quality was achieved, and evaluation of impact. Most case-studies described evaluation of case data while some included vector control or surveillance data. Only two programmes emphasized the quality or coverage of interventions. An important part of M&E is analysis and swift feedback to the periphery, which should theoretically stimulate effective programme response, clearly M&E needs to be undertaken with a spirit of surveillance and response.

Only two case-studies highlighted the results of monitoring and showed that the programme did not achieve the coverage or quality targets. No case-studies included information on M&E of inputs and outputs. The Malaysia case-study gave the most comprehensive results of intervention monitoring, showing the difficulty in achieving coverage and timeliness of case investigation, diagnosis, vector control, and the intensive passenger screening programme.

For other countries, case reporting appeared to be the main tool for evaluation. Bhutan checked weekly case reports from the health facilities. The malaria programme followed up with health facilities for missing or incomplete reports, and if an increase in cases was reported, the respective health center was alerted. Namibia also reviewed weekly case reporting. In recent years, roll out of better diagnostic tools and coverage improved data quality and representation of the malaria burden. In addition, IRS quality was monitored after quality declined. A weakness cited in the Namibia case-study was the lack of data and limited feedback to the sub-national programmes.

Malaysia developed a web-based surveillance database in 2000 for M&E, which facilitated first the reporting of malaria cases, then included case investigation and vector control intervention data which could be monitored. Also in 2000, a separate online case notification system for health providers was introduced, where private and public health facilities rapidly reported all notifiable infectious diseases. The malaria programme regularly reviewed data from both systems and identified and contacted the hospitals, clinics, and private providers that failed to notify cases. The use of two parallel systems could have been cumbersome, but the report stated that the national and state programme officers used both databases to drive management decisions.

The monitoring and evaluation of malaria activities in Sri Lanka was coordinated by the Regional Malaria Officers (at district level) and by the AMC Directorate (national level). Sri Lanka built a web-based case information system in 2009, separate from the national health information system, to ensure reporting within 24 h. The malaria programme planned to integrate the malaria reporting system into the national health information system after reaching elimination. In 2010, the malaria programme introduced a toll free hotline for private sector case reporting. In 2009, to increase data analysis, review and improve the feedback loop to the sub-national programme offices the malaria programme instituted case review meetings. These meetings were attended by Directorate and regional malaria programme officers, where details of each case and follow up measures were reviewed, and they provided an opportunity for feedback to the regional malaria officers, who then relayed information back to the districts. These meetings showed an openness to review, evaluation and change by both the national and regional levels.

There was a lack of information on the tools and processes used in Cape Verde and the Philippines for M&E.

For Turkey and Turkmenistan, resurgences demonstrated the weaknesses in monitoring and evaluation. In Turkmenistan, while transmission decreased when funding was available and activities were well-organized, delays in diagnosis, treatment and reporting fueled the outbreaks of 1998–99 and 2002–03. Case reporting did not flow to the central level in time for evaluation. Similarly, in Turkey, the delayed reporting to the provincial and national level contributed to the 1998–99 outbreak.

### Enabling factors

There are many factors that enable or hinder progress towards elimination for a national malaria programme. Political commitment, funding, and human resources are three key factors that have a large influence on programme progress. These factors also determine the consistency of malaria programme implementation. Table 6 provides key learnings on enabling factors for malaria programmes.

**Table 6:** Key learnings on enabling factors of malaria elimination programmes

• Political commitment at the regional, national, provincial, district, and community levels took many forms and contributed to programme success • Sustained and long-term financial commitment to the malaria programme is a key to success • Consistency of malaria programmes depends on political commitment, human and financial resources, leadership of the malaria programme and operational capacity of the overall health system	

#### Political commitment

Depending on the country’s level of decentralization and its health system structure, political commitment at the national or local level was a driving factor for malaria programme success. Political commitment took many forms, including enacting mandates or laws that support vector control activities and surveillance, ensuring adequate domestic funding and leadership and vision for elimination by national or local level leaders. Turkmenistan provides an example of the deleterious impact of waning political commitment when malaria incidence declines.

In Mauritius, there was a high degree of political commitment at the national level, evidenced by the consistent domestic financing of the programme. National policy was supportive to elimination, contributing to a high level of participation by residents in malaria control activities. There were penalties for non-compliance and health inspectors had legal power to inspect dwellings. Similarly in Malaysia, national policies and legislation were enacted to support elimination; for example, the malaria programme could engage in IRS and ITN distribution on private property and all foreign workers had to undergo screening for malaria before receiving a work permit. Sabah and Sarawak States had additional state-level ordinances to support malaria control activities.

Turkey’s first elimination attempt was successful in part due to steady political commitment. Turkey experienced an epidemic in 1977, then signed the Tashkent Declaration in 2006, which outlined the strategy to achieve elimination in nine countries of the WHO EURO Region by 2015. With the Declaration, the malaria programme had the necessary political support to transition toward elimination in 2008.

The Bhutan malaria programme also had the support of the Ministry of Health—outbreak reports reached the President’s office. Namibia’s elimination programme had the support of the Minister of Health, who was the former manager of the malaria programme and an advocate for regional elimination. In Cape Verde, an increase in malaria mortality (to eight deaths) in 2006 led to a greater level of attention of government leaders and the development of a new elimination goal of 2020.

National-level political commitment for elimination in Turkmenistan waned in the years leading up to the outbreak in Mary Province (2002–2003). In response, commitment was strengthened from 2004 onwards, as evidenced by the government financing dedicated to malaria in preparation for the certification process in 2010. The country then drew up a comprehensive national plan for prevention of reintroduction with support and commitment from the Ministry of Health and Medical Industry, Finance, and intersectoral cooperation.

In other case-studies, commitment at the local level was considered to be a driving factor for success. Malaysia’s Sabah State developed and presented to the national programme a 5 years action plan for malaria control, which built the case for an increase in funding, human resources and development of subsector outpost offices in remote areas, in addition to scaling up of vector control, surveillance and community-based activities. The plan was successful and the state obtained funding from the national government for 100 additional positions. As a result, the proportion of cases investigated increased from 40 to 87 % and malaria offices in remote localities were opened to provide microscopy, vector control, mass blood surveys, and health education.

Philippines also provided an example of the strength of commitment at the local level. Municipalities and communities participated and developed civic duty and pride through malaria programme activities, such as environmental clean-up events. The level of commitment and malaria experience of provincial managers had a large influence on the success of the programme. For example, Apayao and Laguna Provinces were highlighted in the case-study as having strong, knowledgeable, dedicated, well-liked and respected leaders, which drove action and success in their provinces.

Sri Lanka’s malaria programme had commitment to malaria at both national and local levels. The malaria programme and Ministry of Health showed commitment through the maintenance of surveillance and vector control in the conflict zone. Commitment by the Ministry of Health continued even with very low malaria cases, evidenced by the maintenance of specialized malaria screening in health facilities and continuation of entomological surveillance.

#### Stable and predictable funding and human resources

Strong financial commitment has led to strong malaria control programmes. The case-studies showed three examples of resurgence primarily due to a reduction in human and financial resources for malaria elimination. Some countries relied purely on domestic financing, which caused some challenges in the past but overall appeared to increase the consistency of the programme when compared to countries that rely heavily on donor financing. Of interest is the specific question regarding how to maintain funding and skills of IRS spraymen and other staff on needed response activities when cases are very low or reach zero.

Three countries that have successfully eliminated malaria today had experienced resurgences after reaching or nearly reaching elimination in the past, in most part due to declines in funding or human resources. Reduced funding in Mauritius contributed to the resurgence in the 1970s. Inadequate financial and human resources led to Turkey’s 1977 epidemic and again to the 1993–96 epidemic, where there were insufficient staff and health facilities in the area of a major development project, and insufficient malaria expertise at the provincial level. There was a decrease in staffing in receptive and vulnerable areas and delays in identification and reporting of cases. The lack of human and financial resources in Turkmenistan contributed to the 1998–99 outbreak as technical skills and declining staff were lost in the 1990s.

Post-resurgence, these countries built up their programmes in order to achieve elimination. Mauritius, where the government funded the malaria programme, spent over US $2 per capita on malaria in the period after the resurgence, despite zero indigenous transmission, in an effort to minimize the risk of resurgence. Per-capita expenditure during the first elimination attempt, before this latest resurgence, ranged from $6 to $3. A 100-person surveillance team and 100-person vector control team spent nearly 100 % of time on malaria-related activities, a relatively large workforce in a context of zero indigenous cases. After the epidemic of 1977, Turkey increased its allocation to the malaria programme with a corresponding increase in the number of surveillance agents, laboratories, and microscopists. The country found that maintaining skilled personnel was essential to achieve elimination and prevent resurgence through rapid response to outbreaks.

After the 1998–99 outbreak in Turkmenistan, an initiative to build up the number of epidemiologists, lab technicians, and parasitologists began. Then, from 2005 to 2009, in preparation for the elimination goal, the programme doubled the number of malaria staff in parasitology, entomology, and laboratory diagnostics. By 2010, as mentioned previously, 11–20 % of the government malaria budget was spent on training, an indicator of the Ministry’s efforts to maintain quality interventions. The 2009–10 programme costs were covered mainly by the government.

Malaysia has had recent success in maintaining low transmission, partly attributable to the strength of the health system. Malaysia provided one of the few examples of a fully domestic-funded malaria programme in the elimination phase, which has led to a greater degree of consistency in funding and human resources when compared to other, donor-funded programmes.

Bhutan maintained a cadre of malaria technicians, even during years of declining malaria incidence. However, at the time of case-study publication, during elimination, these technicians were to be integrated into multi-purpose health workers. With this transition, there is a risk that commitment to malaria could wane. The case-study also reported on the importance of Global Fund grant funding (Rounds 4 and 7) for staff capacity building, development of community leadership and action, and malaria prevention commodities for hard to reach populations.

In the Philippines, provincial manager empowerment and ownership has increased consistency and increased funding by local governments. The case-study also noted the importance of Global Fund and other donor funding to maintain programme capacity.

Sri Lanka has had an adequate level of funding and resources over time, evidenced by consistent activities and the maintenance of specialized resources such as malaria-only diagnostic centers in health facilities. However, there was concern noted in the case-study about long-term, sustainable funding as the country reached its goal of zero malaria cases, in part because of the historical contributing factors to the 1987 epidemic, which included a shortage of Regional Malaria Officers. In addition, full time malaria programme staff decreased by 29 % from 2004 to 2009, which reduced the number of public health field officers and IRS spraymen who were moved to other positions (e.g., clerks in medical institutions, drivers, assistants) and to other disease priorities (e.g., dengue) and were not easily released to work on malaria. These transfers of positions were likely due to the low number of cases where administrators did not consider it necessary to maintain a malaria control employee.

Cape Verde and Namibia provided examples of inconsistent funding and human resources. Cape Verde has received funding from the Global Fund and the Government of Spain. However, shortages in trained human resources for health and malaria control have continued to constrain the programme, evidenced by the single professional running the entire malaria programme at the national level.

Similarly, human resources and funding in Namibia were not considered adequate, even with the donor funding. There were vacant positions at every level, which led to a reactive rather than a proactive and planned approach. Staff turnover translated to an increased workload.

#### Consistency of programme implementation

Consistency of programme implementation over time depends on the level of political commitment, human and financial resources, and leadership of the malaria programme. It also depends on the operational capacity of the programme and the overall health system structure. Comparing the consistency, coverage and quality of interventions across the countries in the case-studies proved challenging, as these aspects of the programmes were not adequately documented. Changes in leadership, commitment and funding levels led to decreased consistency in some programmes. In three cases, programmes were consistent, leading to major reductions in malaria incidence, which precipitated a lower level of commitment and attention to the malaria programme and a lowering of quality and coverage of the programme. In these cases, resurgence was around the corner, and instigated a rise in programme quality and coverage.

Turkey had success in its first elimination attempt in part due to the experienced, specialized network that carried out operations. Turkmenistan’s outbreak of 1998–99 was partly due to a weakening of the surveillance system. Post-outbreak, the malaria programme ramped up training, surveillance and vector control activities, and ensured the supply of malaria control stocks well after the outbreak was contained.

Success in maintaining low cases in Malaysia, Bhutan and Sri Lanka was partly attributed in the case-studies to the strength of the health system and available infrastructure, including consistent availability of supplies, even in the conflict affected districts of Sri Lanka. However, in Malaysia, the high turnover of leadership in the disease control programme translated to a loss of institutional malaria knowledge and may have impacted the quality of implementation.

Financial and political support in Mauritius allowed for consistency of implementation, even in the POR phase. For example, the passenger screening programme was maintained, which continued to draw human and financial resources during a period of zero local transmission.

The Cape Verde and Namibia case-studies did not report a consistent level of malaria activity implementation. Both programmes had inadequate IRS coverage and quality at times. Restructuring of the programme and health system multiple times in the Philippines led to gaps in coverage of interventions, which affected programme performance. The case-study emphasized the role of the provincial manager in driving programme consistency through ownership, initiative and expertise.

## Discussion

This paper reviewed nine case-studies examining malaria eliminating programmes and found that these programmes operated in highly diverse and challenging ecological, epidemiological, financial, political, and organizational contexts. However, despite this diversity, commonalities and learning points were identified that can help programme managers, policy makers and funders improve the functioning of malaria elimination programmes. Malaria programmes that were successful in eliminating malaria or greatly reducing malaria incidence had the following characteristics: clear lines of accountability and some degree of verticality in the programme; sustained political commitment and funding; a high degree of flexibility and adaptation to changing circumstances and tools; and multi-sector collaborations that facilitated response to threats of outbreak and resurgence.

The analysis of case-studies was limited by several factors. The original case-studies were based on retrospective data collection, and the quality of data varied across topics and themes across the case-studies. Data collection for the case-studies was broad, covering all malaria control strategies and activities and across many decades, meaning that detailed information on programme management strategies was not available for every case-study nor for every year covered in the data collection process. Furthermore, each final case-study report underwent a major review and editing process, which may have introduced errors, deleted concepts or changed perspectives. Due to financial and time constraints, a second round of in-country visits, key informant interviews and record review were not conducted for this cross-case analysis. Because of these limitations, relevant and helpful information or experience from national malaria programmes may not be represented in this analysis.

The comparative analysis distilled experience on the core components of programme management thought to be essential to accelerating towards global eradication. However, the analysis was unable to make specific recommendations on what did or did not lead to country progress towards elimination due to differences in the malaria programmes that exist beyond programme management. What was possible was to determine how malaria programmes handled challenges that were outside of their sphere of influence, partially under the influence of programme, or completely under the influence of the programme.

Malaria programmes faced several obstacles over which they had very little control. A major challenge was operating in a decentralized and integrated health system, where, in most cases, provincial or district health offices controlled malaria resources and implementation without defined, clear roles and responsibilities. The experience and knowledge base at the national programme was no longer utilized as it was in the past, and oftentimes the provincial offices did not have adequate training in the beginning of these transitions. Thus the subnational systems were not able to develop and implement tailored surveillance and response systems that are able to deal with the heterogeneity of transmission that occurs in elimination settings. In an integrated programme, diagnosis and treatment were provided by the general health services, generally by those without malaria expertise. For countries where decentralization or integration will be implemented, evidence suggests that integration must be gradual and well-planned. District officers must have adequate training and supervision and health workers need preparation and motivation to assist with malaria control. If possible, malaria programmes should advocate for sustaining some elements of verticality, such as multipurpose malaria-focused workers, to ensure sufficient attention to malaria. These positions can support non-specialists in the general health system and increase the quality of services [23].

Stronger malaria programmes have clear accountability by identifying who is responsible for achieving elimination, but this clarity can be challenging in an integrated and decentralized context. Experience from other disease eradication programmes found that designating a responsible central unit or an individual ensures leadership and coordination [24, 25]. National and state-level elimination plans are also helpful. A multi-sectoral task force, at the state or provincial level, ensures engagement from ministries and experts from other disciplines [24, 25]. Lastly, malaria programmes should seek to empower and increase authority of local level staff to ensure strong engagement and ownership of the malaria programme. Building management skills at the lower levels is one way to empower staff [26]. Implementing monthly case review meetings, convened by the national programme, where district level officers work together to interpret outcomes and identify lessons to improve daily practice, is a form of organizational learning and supportive supervision.

Malaria programmes have a degree of control over malaria programme capacity, supervision and motivation of workers. The productivity of workers and quality of services provided are critical to programme performance and both rely on staff capacity [26]. In most outbreaks or resurgences documented in the case-studies, contributing factors were inadequate staff, insufficient training and low-quality supervision. Experience from the malaria and smallpox eradication programmes has shown the necessity of adequate personnel that are well-trained, supervised, motivated, and capable of flexible action, evaluation and problem solving [4, 24]. Thus, training programmes must include both technical and operational components; on-the-job training, with emphasis on supervision and coordination [27]. Programmes can also consider shifting resources to continuing education and development, as large investments in training are often lost when there is no maintenance [26, 27]. Continuing education can also improve retention by incentivizing and motivating personnel. Supervision is also critical to programme quality, in particular for decentralized and integrated disease programmes and for those that depend on high quality surveillance activities that are field-based [21, 28]. Good supervision requires updated job descriptions with clear descriptions of roles and responsibilities [29]. While supervision usually takes the form of peripheral visits and checking information and supplies, monthly review meetings are another way to supervise and also facilitate peer-to-peer learning and exchange [24]. Problem solving and feedback, in particular supportive feedback, are important parts of supervision and build worker motivation [21].

The case-studies showed that malaria elimination workers are a diverse group, including paid and volunteer health workers, malaria programme and hospital staff, seasonal employees, and multi-sector partners. These personnel have different values and are motivated by different factors. For paid staff, a basic motivation package should be ensured, including reasonable level and timing of pay, working environments, potential for learning career advancement, and system capacities [26, 27]. Malaria programmes may be able to lobby their Ministry to ensure there is a basic package in place. Malaria programmes can also build forums for professionals to associate and learn from each other, and develop criteria for career advancement based on performance [17]. Perhaps most important, motivation is high when there is a clear goal and endpoint—malaria programmes can clarify their strategy and milestones, bringing the team along with them [24].

Malaria programmes have control over their efforts to improve the level of political commitment and funding, programme strategy and collaborations for malaria elimination. Political commitment at the global and national level is needed for elimination, as very well evidenced by experience from smallpox and polio eradication [24, 29]. Reliable financial commitment is also needed, from both domestic and international sources [2, 23]. Throughout the case-studies, the declaration of a national or subnational elimination goal often sparked and sustained political commitment and increased funding. In some cases, regional or global forums catalyzed support for a national elimination goal, through building awareness and friendly competition amongst countries.

The case-studies did not sufficiently document strategic plan development and their adjustments and adaptation, stratification and targeting of interventions. Targeting requires access to and analysis of real-time information with built-in feedback mechanism to the field where implementation decisions are made. Smallpox eradication experience shows that developing and using a minimum set of indicators will likely improve data use and utility in the field [24]. Targeted implementation and supervision is possible when good quality and real-time information is available [28].

There is a risk that elimination programmes are not flexible enough to adapt to conditions that continuously evolve as a country approaches zero local malaria cases. Instead of a ‘business as usual’ attitude, successful programmes in the case-studies constantly adapted new techniques and tools. Awareness and use of new tools is facilitated by having access to published and grey literature and to forums that bring together countries and partners to share experiences. In addition, strong programmes identified evolving threats to elimination, such as major development projects, and worked with other Ministries and the private sector to minimize the risks. In some cases, effective collaborations can entail contracting out services to private companies or NGOs.

## Conclusion

Global malaria eradication will require well-managed malaria programmes providing high quality implementation of evidence-based strategies, founded upon strong surveillance and response strategies tailored to the subnational level transmission context, with adequate funding and human resources to sustain malaria elimination and prevention of reintroduction. A first step toward achieving this goal is to align national malaria operational plans, broken down to the subnational level, with the current operational and implementation guides, the GTS and the AIM. Roles and responsibilities at each level and across agencies must be clarified, including the multi-sectoral collaborators that will be integral to achieving elimination.

Management of malaria programmes may be enhanced by further training in supervision and management skills. The most technically savvy workers typically lack management experience [24]. An assessment of management practices and skills specific for malaria elimination may be helpful, in addition to workshops on organizational learning, problem solving, and financial management which can build morale in addition to skills [27, 30].

Based on evidence from other disease eradication programmes, there is a need to develop a minimum set of indicators that relate to elimination and eradication goals. Malaria programmes must have well-functioning real-time information systems and capacity for analysis with timely feedback to the field. Programme planning and targeting will not happen without access to good data and analysis.

Lastly, as seen from the gaps in information and evidence provided in the case-studies, it is clear that national operational plans must be accompanied by a portfolio of context-specific, programme management-related operational/implementation research that the programme will use to adapt and adjust strategies and interventions to achieve the highest level of impact. There are still important gaps in evidence, such as how effective supervision is carried out, how workers are best motivated, and how to improve performance and retention of health workers in resource-constrained environments [21]. There are also evidence gaps on incentives and their use, in particular when working with community health workers [26]. Implementation of evidence-based, updated strategies along with building management and supervisory skills will move malaria programmes towards elimination and support steady progress towards global eradication.

## Additional files

10.1186/s12936-016-1518-9 Conceptual framework/data collection matrix (original).
10.1186/s12936-016-1518-9 Bibliography of references for the development of the conceptual framework.

## Abbreviations

AIM: Action and Investment to Defeat Malaria
BTN: Bhutan
CPV: Cabo Verde
GMEP: Global Malaria Eradication Programme
GTS: global technical strategy
IRS: indoor residual spraying
ITN: insecticide-treated net
IVM: integrated vector management
LKA: Sri Lanka
LLIN: long-lasting insecticide-treated bed nets
MUS: Mauritius
MYS: Malaysia
NAM: Namibia
PHL: Philippines
POR: prevention of re-introduction
TKM: Turkmenistan
TUR: Turkey
WHO: World Health Organization
UCSF: University of California, San Francisco
