# Supplementary material for: The central role of national programme management for the achievement of malaria elimination: a cross case-study analysis of nine malaria programmes
Source: Malar J. 2016 Sep 22;15:488. doi: 10.1186/s12936-016-1518-9 (PMC5034437; doi:10.1186/s12936-016-1518-9)
Supplement: Supplementary file 1 — 10.1186/s12936-016-1518-9 Conceptual framework/data collection matrix (original). [file 12936_2016_1518_MOESM1_ESM.pdf]

Conceptual Framework/Data Collection Matrix (original) - PROGRAM MANAGEMENT

	BHUTAN		CAPE VERDE		MALAYSIA		MAURITIUS	
	AVAILABLE CASE STUDY DATA	Additions/Questions	AVAILABLE CASE STUDY DATA	Additions/Questions	AVAILABLE CASE STUDY DATA	Additions/Questions	AVAILABLE CASE STUDY DATA	Additions/Questions
Malaria Programme Status								
**Vertical vs. horizontal chain of command**								
Centralized/Decentralized health system								
Vertical vs. integrated malaria program								
**Effective generic management practices**								
Supply of materials, supply chain								
Leadership of interventions								
Sustained commitment								
Control of resources								
Management information								
Incentives and accountability								
Supervision skills and procedures (monitoring)								
Capacity building/skills training/maintenance								
**Additional information**								
**Application of managerial practices**								
Organization development								
Organizational learning								
Action learning								
Action research								
Behavior change								
Social marketing methods								
Resourcing managerial practices								
**Implications for research**								

NAMIBIA		PHILIPPINES		SRI LANKA		TURKEY		TURKMENISTAN
AVAILABLE CASE STUDY DATA	Additions/Questions	AVAILABLE CASE STUDY DATA	Additions/Questions	AVAILABLE CASE STUDY DATA	Additions/Questions	AVAILABLE CASE STUDY DATA	Additions/Questions	AVAILABLE CASE STUDY DATA

Additions/Questions
