# Supplementary material for: The central role of national programme management for the achievement of malaria elimination: a cross case-study analysis of nine malaria programmes
Source: Malar J. 2016 Sep 22;15:488. doi: 10.1186/s12936-016-1518-9 (PMC5034437; doi:10.1186/s12936-016-1518-9)
Supplement: Supplementary file 2 — 10.1186/s12936-016-1518-9 Bibliography of references for the development of the conceptual framework. [file 12936_2016_1518_MOESM2_ESM.pdf]

Additional File 2. Bibliography of references for the development of the conceptual framework

Accountability

Chan M, Kazatchkine M, Lob-Levy J, Obaid T, Schweizer J, Sidibe M, Veneman A, Yamada T. Meeting the Demand for Results and Accountability: A Call for Action on Health Data from Eight Global Health Agencies. Plos Med. 2010;7:e1000223.

Rowe AK, de Savigny D, Lanata CF, Victora CG. How can we achieve and maintain high-quality performance of health workers in low-resource settings? Lancet. 2005;366:1026-35.

Capacity building and sustainability

Aylward RB, Linkins J. Polio eradication: mobilizing and managing the human resources. Bull of the World Health Organization. 2005;83:268-273.

Bossert TJ. Can they get along without us? Sustainability of donor-supported health projects in Central America and Africa. Soc Sci Med. 1990;30:1015-1023.

Delacollette C, Van der Stuyft P, Molima K. Using communit health workers for malaria control: experience in Zaire. Bull of the World Health Organization. 1996;74:423-430.

Drager S, Gedik G, Poz MRD. Health workforce issues and the Global Fund to Fight AIDS, Tuberculosis and Malaria: an analytical review. Human Resources for Health 2006;4:23.

Dussault G, Dubois C. Human resources for health policies: a critical component in health policies. Human Resources for Health. 2003;1:1-16.

Hongoro C, McPake B. How to bridge the gap in human resources for health. Lancet. 2004;364:1451-1456.

Shediac-Rizkallah MC, Bone, LR. Planning for the sustainability of community-based health programs: conceptual frameworks and future directions for research, practice and policy. Health Education Research. 1998;13:87-108.

Wyss K. An Approach to classifying human resources constraints to attaining health-related Millenium Development Goals. Human Resources for Health. 2004;2:11.

Disease eradication:

Abimbola S, Malik AU, Mansoor GF. The Final Push for Polio Eradication: Addressing the Challenge of Violence in Afghanistan, Pakistan and Nigeria. PlosMed. 2013;10:e1001529.

Aylward RB. Lessons from the Late Stages of the Global Polio Eradication Initiative. “Disease Eradication in the 21st Century: Implications for Global Health”, eds SL Cochi and WR Dowdle.

Aylward RB, Acharya A, England S, Agoocs M, Linkins J. Global health goals: lessons from the worldwide effort to eradication poliomyelitis. The Lancet. 2003;362:909-914.

Bhattacharya S. The World Health Organization and global smallpox eradication. J Epidemiol Community Health. 2008;62:909-912.

Gustavsen K, Hopkins A, Sauerbrey M. Onchocerciasis in the Americas: from arrival to (near) elimination. Parasites and Vectors. 2011;4:1-6.

Henderson DA. Lessons from the eradication campaigns. Vaccine. 1999;17:S53-S55.

Henderson DA. Principles and lessons from the smallpox eradication programme. Bull World Health Organ. 1987;65:535-46.

Hopkins DR, Ruiz-Tiben E. Strategies for dracunculiasis eradication. Bull World Health Organ. 1991;69:533-40.

Loevinsohn B, Aylward B, Steinglass R, Ogden E, Goodman T, Melgaard B. Impact of Targeted Programs on Health Systems: A Case Study of the Polio Eradication Initiative. American Journal of Public Health. 2002;92:19-23.

Makoy S, Becknell SR, Jones AH, Waat G, Ruiz-tiben E, Hopkins DR. Use of surveillance in disease eradication efforts, Part 2: Lessons learned in Guinea worm disease (dracunculiasis) eradication. In: Infectious Disease Surveillance, Second Edition, 2013.

Melgaard B, Creese A, Aylward B, Olive J.M., Maher C, Okwo-Bele JM, Lee JW. Disease eradication and health systems development. Bull of the World Health Organization. 1998;76:26-31.

Mills A. Mass campaigns versus general health services: what have we learnt in 40 years about vertical versus horizontal approaches? Bull World Health Organ. 2005;83:315-6.

Toole M, Simmonds S, Coghlan B, Mojadidi N. Evaluation of the Global Polio Eradication Initiative. 2009; 1-49.

<u>Leadership</u>

Chigudu S, Jasseh M, d'Alessandro U, Corrah T, Demba A, Balen J. The role of leadership in people-centred health systems: a sub-national study in The Gambia. Health Policy and Planning. 2014;1-12.

<u>Malaria</u>

Alonso PL, B. G., Arevalo-Herrera M, Binka F, Chitnis C, Collins F, Doumbo OK, Greenwood B et al. A Research Agenda to Underpin Malaria Eradication. PLoS Med. 2011;8: e1000406.

Bennett A, Avancena ALV, Wegbreit J, Cotter C, Roberts K, Gosling G. Background paper: The Private Sector's Role in Malaria Surveillance. San Francisco: UCSF Global Health Group, 2014.

Birx D, de Souza M, Nkengasong JN. Laboratory challenges in the scaling up of HIV, TB, and Malaria Programs. American Journal of Clinical Pathology. 2009;131:849-851.

Bousema T, Griffin JT, Sauerwein RW, Smith DL, Churcher TS, Takken W, Ghani A, Drakeley C, Gosling R. Hitting Hotspots: Spatial Targeting of Malaria for Control and Elimination. Plos Med. 2012;9:e1001165.

Cao J, S. H., Cotter C, Zhou S, Zhou H, Liu Y, Tang L, Gosling RD, Feachem RGA, Gao Q. Communicating and Monitoring Surveillance and Response Activities for Malaria Elimination: China's "1-3-7" Strategy. Plos Med. 2014;11: e1001642.

Cohen JM, Smith DL, Cotter C, Ward A, Yamey G, Sabot OJ, Moonen B. Malaria resurgence: a systematic review and assessment of its causes. Malaria Journal. 2012;11:1-17.

Cox J. Evaluation of community and health facility based systems for the surveillance of cases of day-3 positive Plasmodium falciparum in Cambodia. Malaria Consortium, 2011.

Feachem RGA, Phillips AA, Hwang J, Cotter C, Wielgosz B, Greenwood BM, Sabot O, Rodriguez MH, Abeyasinghe RR, Ghebreyesus TA, Snow RW. Shrinking the malaria map: progress and prospects. The Lancet. 2010;376:1566-78.

Global Malaria Programme. From Malaria Control to Malaria Elimination: A manual for Elimination Scenario Planning. Geneva: World Health Organization, 2014.

Global Malaria Programme. Global Technical Strategy for Malaria 2016-2030. Geneva: World Health Organization, 2015.

Global Malaria Programme. Malaria Elimination: A field manual for low and moderate endemic countries. Geneva: World Health Organization; 2007.

Gosling J, Case P, Tulloch J, Chandramohan D, Smith Gueye C, Newby G, Wegbreit J, Koita K, Gosling R. Background paper: Program Management Issues in Implementation of Elimination Strategies. San Francisco: UCSF Global Health Group, 2014.

Gosling J, Mintzberg H. The Five Minds of a Manager. Harvard Business Review. 2003;pp 1-10.

Gosling J, Case P, Tulloch J, Chandramohan D, Wegbreit J, Newby G, Smith Gueye C, Koita K, Gosling R. Effective Program Management: A Cornerstone of Malaria Elimination. Am J Trop Med Hyg. 2015;93:135-8.

Heggenhougen HK, Hackethal V, Vivek P. The behavioural and social aspects of malaria and its control. Geneva: World Health Organization on behalf of the Special Programme for Research & Training in Tropical Diseases (TDR), 2003.

Isaacs WN. Taking flight: Dialogue, collective thinking, and organizational learning. Organizational Dynamics. 1993;22:24-39.

Kim L. Crisis construction and organizational learning: Capacity building in catching-up at Hyndai motor. Organizational Science. 1998;9:506-521.

The Malaria Eradication Research Agenda (MalERA). A research agenda for malaria eradication. PLoS Med. 2011;8:1-99.

Mills A, Lubell Y, Hanson K. Malaria eradication: the economic, financial and institutional challenge. Mal Journal. 2008;7:10.1186/1475-2875-7-S1-S11.

Moonen B, C. J., Snow RW, Slutsker L, Drakeley C, Smith DL, Abeyasinghe RR, Rodriguez MH, Maharaj R, Tanner M, Targett G. (2010). "Operational strategies to achieve and maintain malaria elimination." The Lancet 376: 1592-1603.

Najera JA, Gonzalez-Silva M, Alonso PL. Some lessons for the future from the Global Malaria Eradication Programme (1955-1969). PLoS Med. 2011;8:e1000412.

RBM. Action and Investment to Defeat Malaria 2015-2030. Geneva: World Health Organization, on behalf of the Roll Back Malaria Partnership Secretariat; 2015.

RBM. Number 8 Eliminating Malaria: Learning From the Past, Looking Ahead. Geneva: Roll Back Malaria Partnership, October 2011.

Rieckmann KH. The chequered history of malaria control: are new and better tools the ultimate answer? Annals of Tropical Med and Parasitology. 2006;100:647-662.

Sullivan W, Sullivan R, Buffton B. Aligning individual and organizational values to support change. Journal of Change Management. 2010;2:247-254.

Tanner M, Greenwood B, Whitty CJM, Ansah EK, Price RN, Dondorp AM, von Seidlein L, Baird JK, Beeson JG, Fowkes FJI, Hemingway J, Marsh K, Osier F. Malaria eradication and elimination: views on how to translate a vision into reality. BMC Med. 2015;13:10.1186/s12916-015-0384-6.

WHO. The Tashkent Declaration: The move from malaria control to elimination in the WHO European Region. Geneva: The World Health Organization European Region.

Motivation and incentives

Dambisya YM. Discussion paper: A review of non-financial incentives for health worker retention in east and southern Africa. South Africa: University of Limpopo, May 2007. Available: http://www.equinetafrica.org/sites/default/files/uploads/documents/DIS44HRdambisya.pdf

DeBoy JM, Luedtke P, Warren N, Wichman M. Basic Personnel Tools to Help Ensure Future Public Health and Environmental Laboratory Workforce. Public Health Reports. 2010;125:96-101.

Dieleman M, Cuong PV, Anh LV, Martineau T. Identifying factors for job motivation of rural health workers in North Viet Nam. Human Resources for Health. 2003;1:1-10.

Hongoro C, Normand, C. Chapter 71: Health Workers: Building and Motivating the Workforce. Disease Control Priorities in Developing Countries, 2nd edition. Washington DC: World Bank, 2006.

Kurowski C, Mills A. Estimating human resource requirements for scaling up priority health interventions in Low-income countries of Sub-Saharan Africa: A methodology based on service quantity, tasks and productivity. HEFP Working Paper 01/06. London: LSHTM, 2006.

Kurowski C, Wyss K, Abdulla S, Yemadji N, Mills A. Human Resources for Health: Requirements and Availability in the Context of Scaling-Up Priority Interventions in Low-Income Countries. London: LSHTM Health Economics and Financing Programme, January 2003.

Low-Beer D, Afkhami H, Komatsu R, Banati P, Sempala M, Katz I, Cutler J, Schumacher P, Tran-Ba-Huy R, Schwartlander B. Making Performance-Based Funding Work for Health. Plos Med. 2007;4:e219.

Mathauer I, Imhoff I. Health worker motivation in Africa: the role of non-financial incentives and human resource management tools. Human resources for Health. 2006;4: doi 10.1186/1478-4491-4-24.

RBM Case Management Working Group. Diagnostic Testing in the Retail Private Sector: Lessons Learned. London: Roll Back Malaria Case Management Working Group, 2013.

WHO. Improving Health Outcomes of the Poor, Report of Working Group 5 of the Commission on Macroeconomics and Health. Geneva: World Health Organization, 2002.

WHO. The World Health Report. Geneva: The World Health Organization, 2000, 1-206.

<u>Public health systems and structures</u>

Atun RA, Bennett S, Duran A. Policy Brief: When do vertical (stand-alone) programmes have a place in health systems? WHO European Ministerial Conference on Health Systems "Health Systems, Health and Wealth." Estonia, 25-27 June 2008. WHO European Region: p. 1-29.

Hashimoto K, Zuniga C, Nakamura J, Hanada K. Integrating an infectious disease programme into the primary health care service: a retrospective analysis of Chagas disease community-based surveillance in Honduras. BMC Health Services Research. 2015;15:10.1186/s12913-015-0785-4.

Khaleghian P, Gupta MD. Public Management and the Essential Public Health Functions. World Development. 2005;33:1083-1099.

Eds Mills A, Vaughan JP, Smith DL, Tabibzadeh I, Eds. Health System Decentralization: Concepts, issues and country experience. Geneva: World Health Organization, 1990.

Editorial: Kickstarting the revolution in health systems research. The Lancet. 2004;363:1745.

Mogedal S, Stenson B. Disease eradication: friend or foe to the health system? Geneva: World Health Organization, 2000:1-68.

Msuya J. Horizontal and vertical delivery of health services: What are the trade offs? Washington, DC: The World Bank, pp1-28.

Schneider A. Decentralization: Conceptualization and Measurement. Studies in Comparative International Development. 2003;38:32-56.

Travis P, Bennett S, Haines A, Pang T, Bhutta Z, Hyder AA, Pielemeier NR, Mills A, Evans T. Overcoming health-systems constraints to achieve the Millennium Development goals. The Lancet. 2004;364:900-906.

Triesman, D. Defining and Measuring Decentralization: A Global Perspective. Los Angeles: University of California, Los Angeles, 2002:1-38.

Supervision and monitoring

Booman M, Sharp BL, Martin CL, Manjate B, la Grange JJ, Durrheim DN. Enhancing malaria control using a computerized management system in southern Africa. Malaria Journal.2003;2:1-5.

Bosch-Capblanch X, Garner P. Primary health care supervision in developing countries. Trop Med Int Health. 2008;13:369-83.

Bryce J, Roungou JB, Nguyen-Dinh P, Naimoli JF, Breman JG. Evaluation of national malaria control programmes in Africa. Bulletin of the World Health Organization. 1994;72:371-381.

The World Bank Independent Evaluation Group. Designing a results framework for achieving results: A how-to guide. Washington DC: The World Bank, 2012.

Supply chain

Pluess B, Mueller I, Levi D, King G, Smith TA, Lengeler C. Malaria – a major health problem within an oil palm plantation around Popondetta, Papua New Guinea. Malaria Journal. 2009;8:10.1186/1475-2875-8-56.

Training

Ofori-Adjei D, Arhinful DK. Effect of training on the clinical management of malaria by medical assistants in Ghana. Soc Sci Med. 1996;42:1169-1176.

Ssekabira U, Bukirwa H, Hopkins H, Namagembe A, Weaver MR, Sebuyira LM, Quick L, Staedke S, Yeka A, Kiggundu M, Schneider G, McAdam K, Wabwire-Mangen F, Dorsey G. Malaria case management after integrated team-based training of health care workers in Uganda. Am J Trop Med Hyg. 2008;79:826-833.
